# Supplementary material for: A Lifelike guided journey through the pathophysiology of pulmonary hypertension—from measured metabolites to the mechanism of action of drugs
Source: Front Cardiovasc Med. 2024 May 23;11:1341145. doi: 10.3389/fcvm.2024.1341145 (PMC11153715; doi:10.3389/fcvm.2024.1341145)
Supplement: Supplementary file 2 [file Presentation2.pdf]

1) Upload and generate annotations

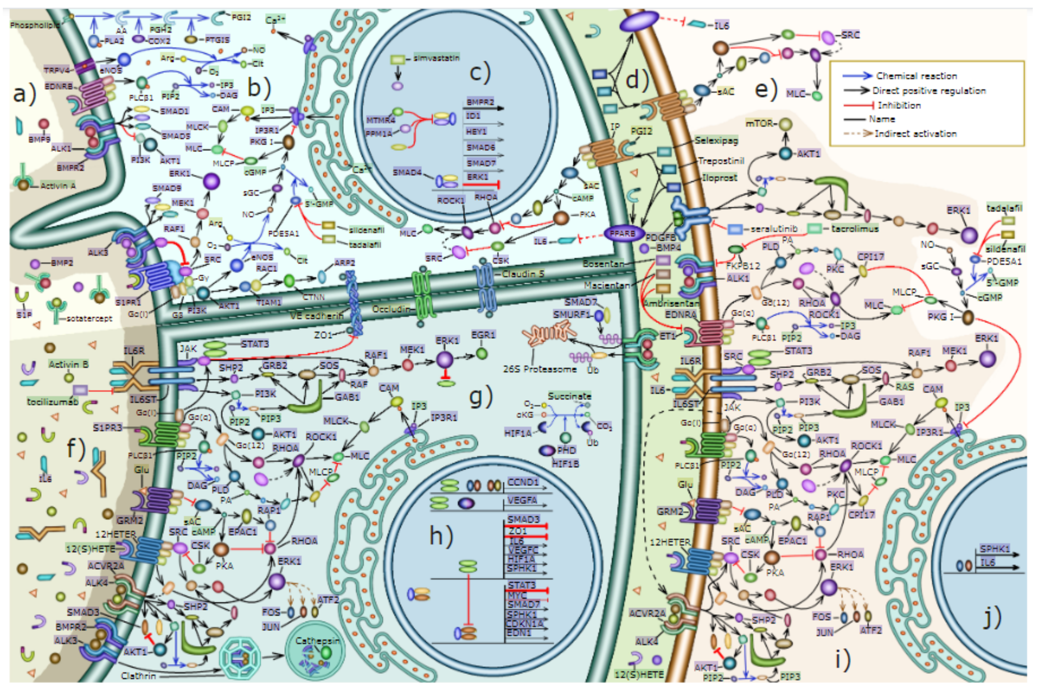

2) Explore the annotations using word clouds

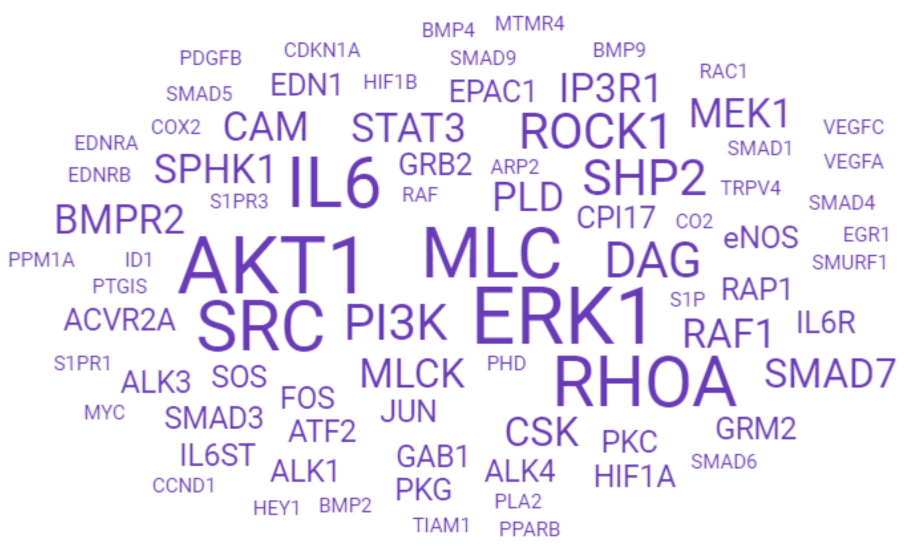

Genes found in the figure

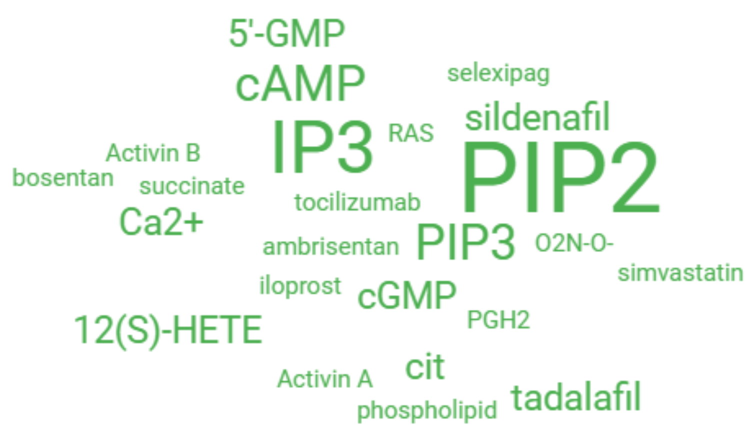

Chemicals that appear on the figure

3) Use the genes to create an enrichment table

interaction
figure genes

4) Find relevant biological processes through statistical enrichment.

Biological Process — Table | Graph | Clustergram | Cloud

Search:

GOID	GO Term	Associated genes	q-value
30509	**BMP signaling pathway**	BMPR2, ACVRL1, SMAD4, ACVR2A, SMAD6, SMAD3, MAPK3, SMURF1, BMP2, SMAD1, ID1, BMPR1A, GDF2, EGR1, SMAD5, SMAD7, SMAD9, BMP4	8.91e-21***
7179	**transforming growth factor beta receptor signaling pathway**	RHOA, ACVRL1, SMAD4, SRC, MTMR4, SMAD3, SMAD6, JUN, SMURF1, SMAD1, ID1, BMPR1A, FOS, SMAD5, SMAD7, SMAD9	1.19e-15***

This tells us that BMP signaling is important.

Biological Process — Table | Graph | Clustergram | Cloud

Search: endothelial

GOID	GO Term	Associated genes	q-value
1938	**positive regulation of endothelial cell proliferation**	ARNT, HIF1A, BMPR2, ACVRL1, AKT1, PDGFB, JUN, BMP2, GDF2, VEGFC, VEGFA, PIK3CD, BMP4	1.27e-13***
10595	**positive regulation of endothelial cell migration**	BMPR2, EDN1, AKT1, PIK3CG, PIK3CB, GPLD1, RAC1, VEGFA, PIK3CD, BMP4	1.09e-9 ***
48010	**vascular endothelial growth factor receptor signaling pathway**	RHOA, ROCK1, SRC, PIK3CB, RAC1, VEGFC, VEGFA, PIK3CA	5.69e-7 ***
43536	**positive regulation of blood vessel endothelial cell migration**	HIF1A, NOS3, AKT1, PDGFB, VEGFC, VEGFA	3.60e-5 ***
10575	**positive regulation of vascular endothelial growth factor production**	ARNT, HIF1A, PTGS2, IL6, IL6ST	5.55e-5 ***

Endothelial cell proliferation and migration are affected by these genes.

Biological Process — Table | Graph | Clustergram | Cloud

Search: smooth muscle cell

GOID	GO Term	Associated genes	q-value
48661	**positive regulation of smooth muscle cell proliferation**	EDN1, AKT1, PTGS2, IL6R, S1PR1, IL6, PDGFB, BMP4	2.73e-7***
1904707	**positive regulation of vascular associated smooth muscle cell proliferation**	JUN, EDN1, PDGFB, BMPR1A	7.55e-3**

These genes promote smooth muscle cell proliferation.

Biological Process — Table | Graph | Clustergram | Cloud

Search: pressure

GOID	GO Term	Associated genes	q-value
3100	**regulation of systemic arterial blood pressure by endothelin**	RHOA, NOS3, EDN1	3.62e-4***
8217	**regulation of blood pressure**	ACVRL1, NOS3, EDNRA, PTGS2, EDNRB	2.36e-3**

Processes associated with blood pressure control

5) The word cloud asssciiatedd with the enrichment table also contains valuable information

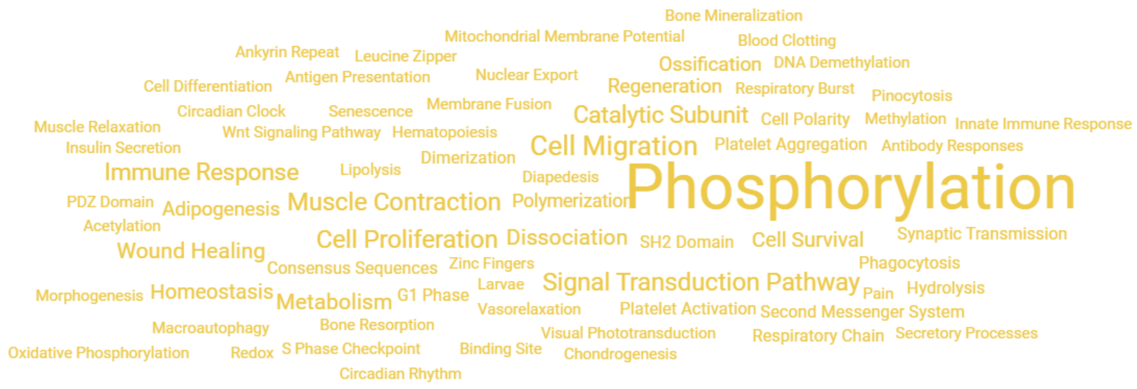

The phenomena associated with the genes on the enrichment table

After Phosphorylation, Signal Transduction Pathway, Cell Migration, Muscle Contraction, Cell Proliferation, and Immune Response, stand out. If we look closely, we can also see Vasoconstriction, Blood Clotting, Platelet Activation, Ossification, and G1 phase. All these phenomena are important for pulmonary hypertension.

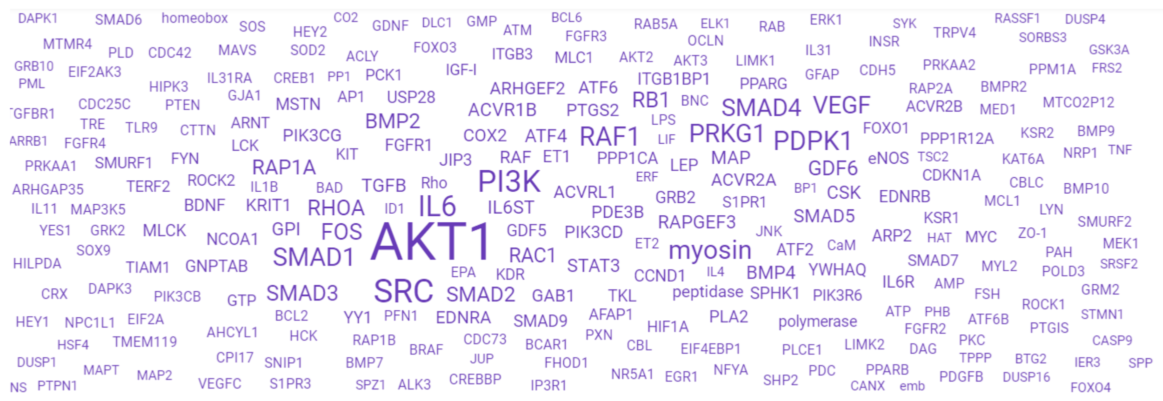

Genes present in the description of the enrichment table.

6) The genes names present in the description of the genes can be used to create a larger enrichment table and reach additional information.

interaction(1
)

Created by Lifelike
lifelike.bio
