# Supplementary material for: A Lifelike guided journey through the pathophysiology of pulmonary hypertension—from measured metabolites to the mechanism of action of drugs
Source: Front Cardiovasc Med. 2024 May 23;11:1341145. doi: 10.3389/fcvm.2024.1341145 (PMC11153715; doi:10.3389/fcvm.2024.1341145)
Supplement: Supplementary file 4 [file Datasheet1.pdf]

Supplementary Material

1 PATHWAYS IN DETAIL

1.1 G protein-coupled receptor signaling

1.1.1 In healthy people S1PR1 activity improves endothelial barrier function, promotes vasodilation, and enables normal PAEC proliferation.

In endothelial cells, S1PR1 is abundant, while S1PR2 and S1PR3 are present at a lower concentration Cantalupo et al. (2017); Wilkerson and Argraves (2014). When S1P binds S1PR1, it leads to the formation of a protein complex that includes ARRB1,2, Gi/0α, Giβ, and Giγ that causes the sequential recruitment, phosphorylation, and activation of SRC, RAF1, MEK1, MEK2, ERK1, and ERK2 Pyne and Pyne (2017); Jain et al. (2018). After that, ERK signaling promotes PAEC and PASMC proliferation by inducing Cyclin D1 expression. Cyclin D1 binds CDK4 or CDK6 to form a complex that is necessary for cell cycle progression through the G1 phase, and the G1/S transition. ERK1,2 is sufficient and necessary for the expression of Cyclin D1 Lavoie et al. (2020). ERK indirectly activates the transcription of the AP-1 components JUN and FOS, and the related protein ATF2. The Cyclin D1 promoter contains a consensus AP-1 site that is activated by FOS, JUN, and a cyclic AMP-response element (CRE) that can be activated by JUN and ATF2 Klein and Assoian (2008). Further, the Cyclin D1 promoter contains three Ets binding sites, ETS1 is activated by ERK1/2 and promotes bovine retinal endothelial cell proliferation Klein and Assoian (2008); Watanabe et al. (2004). A normal plasma concentration of S1P (0.1 to 0.8 µM) prevents endothelial barrier permeability Li et al. (2021). S1P-bound S1PR1 recruits Giβ and Giγ that bind and activate PI3K. Then, PI3K generates PIP3 which recruits AKT1 to the cell membrane where it is activated. Subsequently, active AKT1 recruits the guanine exchange factor for RAC1(TIAM1). Later, AKT1 and TIAM1 bind SIPR1 forming a complex that activates RAC1. After that, RAC1 increases PI3K activity forming a positive feedback mechanism. Lastly, RAC1 inhibits the formation of RHOA-dependent stress fibers and recruits CORTACTIN to actin-nucleation complexes that contain ARP2 and ARP3, this causes the polymerization of actin filaments and EC spreading, therefore improving endothelial barrier function Wilkerson and Argraves (2014). Additionally, AKT-1 activates eNOS by phosphorylating it on serine 1177. After that, eNOS catalyzes a reaction that converts L-arginine and O_2 into L-citrulline and NO, this increases endothelial NO production and secretion. Then, NO activates sGC, which converts GTP to cGMP. Later, cGMP activates PKG, which inhibits Ca^{2+} import Yao et al. (2000), promotes Ca2+ storage in the sarcoplasmic reticulum by inhibiting IP3R1 and activating SERCA Lincoln et al. (2001). Furthermore, PKG I induces MLCP function, which together with a decrease in cytosolic Ca2+ prevents MLC activation and VSMC contraction Toxvig et al. (2019); Lincoln et al. (2001).

1.1.2 S1PR3 signaling promotes vasoconstriction and increases blood vessel permeability.

An elevated plasma concentration of S1P (5 µM) promotes vasoconstriction and vascular permeability. ECs and vascular SMCs express S1PR2, and S1PR3, however, both are far more prevalent in vascular SMCs. After S1P binds and activates S1PR2, or S1PR3, it signals through Gi/0, G12, G13, and Gq Li et al. (2021). G12, and G13 signaling induces vasoconstriction by activating guanine nucleotide exchange factors (GEFs) that transform inactive GDP-RHOA into GTP-RHOA, which activates the Rho-kinases ROKI and ROKII. After that, ROK can directly phosphorylate MLC, and CPI-17, which inhibits MLCP and thus indirectly increases MLC activity Watterson et al. (2005); Somlyo and Somlyo (2003). Furthermore,

G12, and G13 can also activate the enzyme PLD that stimulates the hydrolysis of phosphatidylcholine resulting in phosphatidic acid (PA) production Natarajan et al. (1994). Then, PA is dephosphorylated and transformed into diacylglycerol (DAG), which promotes sustained protein kinase C (PKC) activity. PKC then phosphorylates and activates CPI-17 which increases MLC activity by inhibiting MLCP Somlyo and Somlyo (2000); Watterson et al. (2005). S1PR3-activated Gi/0, Gq, G12, and G13 can stimulate phospholipase C (PLC) Murakami et al. (2010); Watterson et al. (2005); Li et al. (2021), however PLC activation is primarily mediated by Gq Taha et al. (2004). PLCβ with Ca2+ as a cofactor catalyzes a reaction that transforms phosphatidylinositol 4,5-bisphosphate (PIP2) into inositol 1,4,5-trisphosphate (IP3) and DAG Caricasole et al. (2000). As mentioned above, DAG promotes MLC activity. Additionally, when IP3 binds its receptor IP3R1 on the sarcoplasmic reticulum membrane (or the endoplasmic reticulum in ECs), it promotes the release of Ca^{2+} into the cytoplasm that binds and activates Calmodulin (CAM). After that CAM activates MLCK Amberg and Navedo (2013), which phosphorylates and activates MLC, and that leads to VSMC contraction53.

1.1.3 GRM2 and GRM3 signaling inhibits adenylate cyclase activity.

L-glutamate is involved in the endothelial response to oxidative damage and regulates endothelial barrier function Julio-Pieper et al. (2011). The type II metabotropic L-Glu receptors GRM2 and GRM3 are expressed in rat pulmonary endothelial cells Gill et al. (2000). GRM2 and GRM3 are Gi/o-coupled receptors that inhibit Adenylate cyclase (AC) function leading to a lower concentration of cAMP, less PKA and C-terminal Src kinase (CSK) activity, causing an increase in SRC activity Trepanier et al. (2013). The decrease in cAMP hinders cAMP-inducible guanine-exchange factor for Rap (EPAC) function. EPAC enhances endothelial barrier function by increasing cortical actin and activating RAP1, which obstructs RHOA activation Cullere et al. (2005). Additionally, PKA phosphorylates and inhibits MLCK in cultured bovine pulmonary arterial endothelial cellsVerin et al. (1998). Further, PKA promotes vasodilation by inhibiting the IP3-dependent release of Ca^{2+} from the sarcoplasmic reticulum Lucas et al. (2000). Therefore, suppressing AC function promotes MLC-mediated cell contraction Cullere et al. (2005).

1.1.4 12HETER signaling promotes cell proliferation.

12-(S)-HETE binds and activates the G protein-coupled receptor 12HETER (GPR31) that signals through Gi/0 to activate SRC, ERK1 and ERK2 Guo et al. (2011). Furthermore, 12(S)-HETE promotes PASMC proliferation through the activation of ERK Preston et al. (2006). Moreover, 12(S)-HETE causes EC retraction through the phosphorylation of cytoskeletal proteins in a path mediated by PKC Tang et al. (1993). Additionally, 12(S)-HETE leads to MLC2 phosphorylation to induce lymphatic endothelial barrier breaching, this process is mediated by Gα12/13 RHOA and ROCK Nguyen et al. (2016).

1.2 Interleukin 6 Signaling

1.2.1 IL6 signaling initiation.

In PH patients, SRC activity increases the expression of IL6 Zhang et al. (2021); Singer et al. (2011). The IL6 receptor IL6R is absent from the cell membrane of PAECs. However, in the plasma of PH patients, IL6 binds the soluble form of its receptor (sIL6R), and then both bind the coreceptor IL6ST (gp130), which is expressed by PAECs, forming a hetero-hexamer complex that initiates trans IL6 signaling. Under PAH conditions, PASMCs express both IL6, its receptor IL6R and the coreceptor IL6ST, therefore in PASMCs classical IL6 signaling is activated autocrinally Simpson et al. (2020); Pullamsetti et al. (2018).

1.2.2 ERK1, ERK2, and STAT3 are activated by IL6 and promote PAEC and PASMC proliferation.

IL6 promotes PAEC and PASMC proliferation by allowing cell cycle progression during the G1 phase and through the G1/S transition. The complex formed by IL6, IL6R, and IL6ST binds JAK, and either SHP2 Eulenfeld et al. (2012) or SRC to activate RAF/MEK/ERK signaling (Figure 4 a) and STAT3 (Figure 4 b) Birukova et al. (2016); Huang et al. (2014). ERK1 and ERK2 increase the expression of the transcription factors FOS, JUN, and ATF2, which together with STAT3, bind to the promoter of CNND1 and increase the expression of Cyclin D1. Then, CDK4 or CDK6 and CyclinD1 form a complex that allows cell cycle progression through transcriptional regulation Klein and Assoian (2008); Watanabe et al. (2004) (Supplementary section 1.1.1.). Moreover, STAT3 activates the expression of MYC, PIM1, and PIM2 that also control the G1 to S transition Hirano et al. (2000). Additionally, IL6 induces PASMC proliferation through M2 macrophage polarization. IL-6 promotes the differentiation of Th17 cells that secrete IL-17. CD4+ T cells including Th17 cells respond to IL-6 and IL-17 by secreting IL-21, which promotes M2 macrophage polarization. IL-21 and M2 macrophage markers were upregulated in the lungs of patients with idiopathic PAH. IL-21 receptor deficiency in mice leads to hypoxia-induced PH and prevents the accumulation of M2 macrophages in the lungs Hashimoto-Kataoka et al. (2015). Lastly, the cytokines secreted by hypoxic M2 macrophages promote the proliferation of PASMCs Vergadi et al. (2011).

1.2.3 IL6-activated SRC and STAT3 increase endothelial barrier permeability.

After being activated by IL6 signaling, SRC and STAT3 increase endothelial permeability. SRC directly phosphorylates VE-cadherin, this phosphorylation is necessary for VEGFA and VEGFR2-induced endothelial permeability Wallez and Huber (2008) and is involved in IL6-induced VE-cadherin internalization and loss of barrier function in PAECs Birukova et al. (2016). Exposure to IL6 causes an increase in barrier permeability that lasts more than 24 hours. However, the effect of SRC, and VE-phosphorylation on endothelial barrier function is necessary only during the initial hours implying that IL6 increases endothelial permeability through other molecular mechanisms Alsaffar et al. (2018). IL6 reduces the expression of VE-cadherin Alsaffar et al. (2018). Further, IL6-induced STAT3 inhibits the expression of the tight junction components ZO1 and Occludin (Figure 3d, 3f) Yun et al. (2017). Furthermore, STAT3 activates the transcription of IL6, SPHK1, HIF1A, VEGFC, and VEGFA (Figure 3f).

1.2.4 IL6 signaling and hypoxia activate the transcription of VEGFA.

Under hypoxic conditions, the ubiquitination and degradation of HIF1A is inhibited. Then, HIF1A forms a complex with HIF1B (Figure 3e) that binds the VEGFA promoter. When both STAT3 and HIF1A/HIF1B bind the VEGFA promoter, the expression of VEGFA is very high Jung et al. (2005). This constitutes an important connection between hypoxia, inflammation, and increased endothelial permeability that partially explains how IL6 contributes to the development of PH caused by hypoxia Steiner et al. (2009). VEGFA has two effects that are important during PH progression, it promotes angiogenesis, and it increases endothelial barrier permeability through the phosphorylation of junction components Kevil et al. (1998), including ZO1, Occludin Antonetti et al. (1999), and VE-cadherin. The downregulation of VE-cadherin is initiated when VEGFA binds neuropilin 1 (NRP1) Domingues and Fantin (2021) and VEGFR2 that activate SRC. Then SRC phosphorylates the guanine nucleotide-exchange factor VAV2, which activates the small GTPase RAC. After that, RAC activates p21-activated kinase (PAK) leading to the phosphorylation of a highly conserved motif within the intracellular region of VE-cadherin. This causes VE-cadherin to recruit ARRB2 promoting the internalization of VE-cadherin in clathrin-coated vesicles Gavard and Gutkind (2006).

1.2.5 IL6 inhibits apoptosis through AKT signaling.

In cultivated human vascular endothelial cells (HUVECs) AKT phosphorylation requires sIL6R, JAK, and PI3K Zegeye et al. (2018). The mechanism that allows IL6 signaling to activate PI3K is not fully known. However, it involves SHP2, IL6ST, and GAB1 Eulenfeld et al. (2012). IL6-activated AKT inhibits PAEC hyperoxia-induced apoptosis through the inhibition of the proapoptotic member of the Bcl-2 family BAX (Figure 3c) Kolliputi and Waxman (2009). Additionally, AKT stimulates the expression of the inhibitor of apoptosis Survivin in endothelial cells Papapetropoulos et al. (2000).

1.3 TGF signaling.

TGFβ signaling is initiated when a TGFβ family ligand dimer associates with two homodimers, one formed by type II -TGFβ receptor (TβRII) and the second comprising type I -TGFβ receptor (TβRI) Groppe et al. (2008); Tzavlaki and Moustakas (2020). TGFβ ligand binding causes TβRIIs to phosphorylate four threonine or serine residues in the GS domain (TTSGSGSG motif) of TβRIs resulting in the activation of TβRIs Hata and Chen (2016). The activated TβRIs can recruit, phosphorylate, and activate receptor-regulated SMADS (R-SMADs) Hata and Chen (2016). After that, Common partner SMADs (SMAD4) bind two phosphorylated R-SMADs forming a complex that is transported from the plasma membrane into the nucleus where it regulates the transcription of several target genes. After TGFβ ligands activate TβRIIs and TβRIs, the latter phosphorylate and activate SMADs. Additionally, active TβRIIs and TβRIs modulate p38, JNK, AKT, ERK, and RHO signaling Zhang (2009). The mechanism that allows TGFβ ligands to activate ERK signaling requires the following steps: Ligand binding causes the phosphorylation of TβRs that associate with a docking protein like SHP2, SRC, or SHCA. The docking protein then recruits a complex formed by GRB2 and SOS from the cytoplasm to the plasma membrane. Subsequently, SOS activates RAS by catalyzing the exchange of GDP for GTP. After that, GTP-bound RAS binds RAF leading to the sequential activation of RAF, MEK and ERK Zhang (2009) (Figure 5a). After a ligand from the BMP or Activin branch of the TGFβ family associates with them, TβRIIs and TβRIs can bind PI3K, which phosphorylates and activates AKT Zhang (2009) (Figure 5a). AKT activity has several functions which are relevant for PH, it activates eNOS that increases NO production Chang et al. (2011). Also, AKT inhibits BAD and BAX-mediated apoptosis Papapetropoulos et al. (2000). Under certain conditions, TGFβ signaling can also inhibit AKT signaling. One possible molecular mechanism for this inhibition involves the docking protein SHCA, which reduces the expression of AKT and eNOS Abou-Jaoude et al. (2018). Additionally, in hematopoietic cells, TGFβ can reduce AKT signaling activity through the Smad-dependent expression of the lipid phosphatase SHIP Valderrama-Carvajal et al. (2002). TGFβ signaling is modulated through a diverse group of molecular mechanisms Miyazawa and Miyazono (2017). LEFTYA and LEFTYB function as competitive inhibitors for TGFβ ligands Kosaki et al. (1999). Additionally, certain ligands that have a higher binding affinity for TβRIIs prevent other ligands from associating with them Aykul and Martinez-Hackert (2016). BAMBI prevents the formation of functional receptor complexes by associating with TβRIs and TβRIIs. Furthermore, BAMBI collaborates with SMAD7 to prevent R-SMAD activation Yan et al. (2009). SMAD6, and SMAD7 are inhibitory SMADs (I-SMADs) that bind TβRIs preventing R-SMAD activation. Additionally, I-SMADs recruit ubiquitin E3 ligases, like Smad ubiquitin regulatory factors (Smurfs), which mediate the ubiquitination and degradation of active R-SMADS, TβRIs, and TβRIIs. Moreover, Smurfs and Salt-inducible kinase (SIK) enhance the interaction between I-SMADs and TβRIs to reduce R-SMAD activation. Furthermore, I-SMADs associate with active R-SMADs and prevent R-SMADs from associating with SMAD4. Also, I-SMADS present in the nucleus inhibit the transcription of R-SMAD-regulated genes Miyazawa and Miyazono (2017). What's more, the phosphatase PPM1A deactivates R-SMADs and promotes proteosome-mediated degradation of R-SMADs Kokabu et al. (2010).

Lastly, the phosphatase MTMR4 dephosphorylates and deactivates R-SMADS within early endosomes Yu et al. (2010).

1.3.1 Activin signaling is overactivated in PH patients, promotes excessive vascular cell proliferation, and downregulates BMP signaling.

The TGFβ family ligands activin A, and activin B initiate activin signaling. The concentration of activin A is more elevated than normal in PH patients and is significantly correlated to mortality 99. Additionally, pulmonary microvascular endothelial cells (PMECs) isolated from idiopathic PAH patients exhibit augmented activin A expression Ryanto et al. (2021). Moreover, the PAECs of the same group of patients overexpressed activin B Ryanto et al. (2021). Further, the concentration of phosphorylated SMAD2 and SMAD3, and the immunoreactivity of ACVR2B, ALK2, ALK4, and ALK5 was more elevated than normal in PH patients Guignabert et al. (2023). Activin A and activin B associate with high affinity to the TβRIIs ACVR2A, ACVR2B, and BMPR2 Aykul and Martinez-Hackert (2016), which are expressed in human pulmonary microvascular ECs Benn et al. (2017).

1.3.1.1 Normally SMAD-dependent activin signaling inhibits cell proliferation, increases the production of S1P, and modulates Activin and BMP signaling.

The TβRIs ALK4, ALK2, ALK3 and ALK6 compete to bind ACVR2A on the cell membrane Szilágyi et al. (2022). Moreover, activin A and ALK4 are expressed in pulmonary endothelial cells Samitas et al. (2013) and the complex formed by ACVR2A and ALK4 transduces activin A signaling to activate SMAD2 and SMAD3 Szilágyi et al. (2022); Ten Dijke et al. (1994). SMAD2 and SMAD3 bind SMAD4 forming complexes activate the transcription of CDKN1A (p21) and inhibit the expression of MYC to prevent the proliferation of cultured human umbilical vein endothelial cells (HUVECs) Kaneda et al. (2011); Yagi et al. (2002). Additionally, SMAD2 and SMAD3 increase the production of S1P by activating the expression of SPHK1 in rat PASMCs, the increase in S1P production is normally beneficial Wang et al. (2018). Also, SMAD2 and SMAD3 modulate Activin and BMP signaling by stimulating the transcription of SMAD7 von Gersdorff et al. (2000). Further, the complex formed by SMAD2, SMAD3 and SMAD4 can activate the transcription of SMAD3 forming a positive feedback mechanism that amplifies canonical activin signaling Makino et al. (2017).

1.3.1.2 SMAD-independent activin signaling promotes EC proliferation, inhibits apoptosis, and downregulates BMP signaling.

Activin also signals through SMAD-independent pathways. Activin A induces ERK signaling in fibroblasts Jiang et al. (2021). Also, activin B activates ERK signaling in adipose Zhang et al. (2017) and bone marrow-derived Zhang et al. (2014b) mesenchymal stem cells, which are closely related to ECs through the endothelial-to-mesenchymal transition. Further, ERK signaling stimulates PAEC and PASMC proliferation (Supplementary section 1.2.2.). Activin A also causes the sequential activation of PI3K, AKT and eNOS in endothelial cells from the atrioventricular canal during early development Chang et al. (2011). Additionally, AKT signaling inhibits PAEC apoptosis (Supplementary section 1.2.5.). Notably, AKT directly associates with SMAD3 preventing its TβRI-mediated phosphorylation and nuclear translocation Conery et al. (2004); Zhang (2009). Activin signaling promotes the internalization and degradation of BMPR2. When activin A binds BMPR2 it leads to clathrin-mediated endocytosis of BMPR2. Then the vesicles containing BMPR2 fuse with lysosomes that contain Cathepsins and degrade BMPR2 resulting in a lower concentration of BMPR2 in the membrane of in PAECs Ryanto et al. (2021).

1.3.2 The effect of BMP signaling on PH.

The BMP branch of the TGFβ family of ligands includes BMP2, BMP4, BMP6, BMP9, and BMP10. Normally, BMP9 and BMP10 are present in the blood serum at a sufficient concentration to elicit BMP signaling (0.5–15 ng/mL). BMP2, BMP4, and BMP6 are present at a low concentration and their expression is carefully regulated spatially and temporally leading to the formation of dynamic gradients of these ligands. Notably, some endothelial cells express BMP2, BMP4, and BMP6 Kulikauskas and Bautch (2022). Extracellular antagonists including Chordin, Noggin, Gremlin, and MGP modulate BMP signaling. Noggin and Gremlin bind and inhibit BMP2, BMP4, and BMP7, while Chordin binds and inhibits BMP4 Kulikauskas and Bautch (2022).

1.3.2.1 BMP2 signaling is inhibited in PH patients.

Normally, BMP2 signaling prevents excessive pulmonary vascular cell proliferation, preserves endothelial barrier function, and promotes vasodilation by deactivating SRC and stimulating the expression of eNOS. In a mouse model of hypoxia-induced PH, the expression of BMP2 and BMP4 is upregulated in the peripheral pulmonary vasculature after exposure to hypoxia. Additionally, heterozygous null Bmp2+/- mice develop more severe PH than wild-type mice under hypoxic conditions Anderson et al. (2010). BMP2 mostly associates with the TβRII BMPR2, which is normally expressed in PAECs and to a lesser degree in PASMCs Frank et al. (2008). Heterozygous Bmpr2 mutations are common in familial PAH patients. In mice, EC-specific Bmpr2 deletion spontaneously causes PH, and increases the severity of hypoxia-induced PH Anderson et al. (2010); Frank et al. (2008). The TβRI with the highest BMP2 binding affinity is ALK3 (BMPR1A) Heinecke et al. (2009) and the formation and activation of the complex composed of BMP2, BMPR2 and ALK3 leads to the phosphorylation and activation of SMAD1, SMAD5, and SMAD9 Kulikauskas and Bautch (2022). The expression of BMPR2 and ALK3 is markedly reduced in the pulmonary vasculature of PH patients El-Bizri et al. (2008). BMP2 has several beneficial effects that reduce the severity of PH. During the progression of hypoxia-induced PH, the activity of SMAD1, SMAD5, and SMAD9 increases. Then, the BMP-activated SMADs induce the transcription of the inhibitors of differentiation ID1, and ID3, that limit excessive PAEC and PASMC proliferation Lowery et al. (2010). Additionally, SMAD1, SMAD5, and SMAD9 stimulate the transcription of SMAD6 and SMAD7, this is one of the molecular mechanisms that allows precise special and temporal modulation of TGFβ signaling de Vinuesa et al. (2016); Beets et al. (2016). Moreover, BMP-activated SMADs inhibit the expression of ERK1 and ERK2, this is an important step during the endothelial-to-hematopoietic transition and inhibits vascular cell proliferation. Inhibition of SMAD1 and SMAD5 or overexpression of ERK1 caused artery enlargement and reduced the number of hematopoietic stem cells that form in 24 hpf Zebrafish embryos Zhang et al. (2014a). Furthermore, SMAD1 and SMAD5 induce the transcription of HEY1 Beets et al. (2016), which is an important component of the molecular mechanism that regulates the endothelial-to-mesenchymal transition Yu et al. (2021) and pulmonary fibrosis Hardie et al. (2007), two EndMT-related and important processes for PH Ranchoux et al. (2015); Tsutsumi et al. (2019). BMP2 activates ERK phosphorylation. Then, ERK1 deactivates GSK3β preventing the inhibition of β-catenin, which promotes PAEC proliferation and inhibits excessive PAEC apoptosis de Jesus Perez et al. (2009). Additionally, BMP2 signaling preserves endothelial barrier function and promotes vasodilation by activating eNOS Anderson et al. (2010); Coggins and Bloch (2007). Moreover, BMP2 signaling prevents excessive pulmonary vascular cell proliferation by downregulating SRC activity. BMP2 activates BMPR2 and that decreases SRC phosphorylation at Tyrosine 418 in PASMCs Wong et al. (2005).

1.3.2.2 BMP4 inhibits PASMC apoptosis.

In contrast to BMP2 and BMP9, the effects of BMP4 signaling are mostly detrimental to PH patients. The expression of BMP4 is upregulated in the peripheral pulmonary vasculature of mice after exposure to hypoxia. Additionally, heterozygous null Bmp4+/- mice develop less severe PH than wild-type mice under hypoxic conditions Anderson et al. (2010). BMP4 associates with the TβRII BMPR2 and the TβRI ALK3 leading to the phosphorylation and activation of SMAD1, SMAD5, SMAD9 and PI3K Anderson et al. (2010); Wu et al. (2014). In human PASMCs BMP4 signaling inhibits the cleavage of procaspase-3 and the expression of caspase-3, these BMP4 effects require AKT activity. Additionally, ALK3 deletion in mice PASMCs prevented the muscularization of distal pulmonary vessels caused by hypoxia El-Bizri et al. (2008). Therefore, BMP4 signaling contributes to the excessive PASMC cell proliferation observed in PH patients Wu et al. (2014). Further, BMP4 causes vascular calcification Yang et al. (2020); Ye et al. (2023), and promotes the differentiation of endothelial cells into osteoblasts Yu et al. (2021).

1.3.2.3 BMP9 promotes EC quiescence, inhibits apoptosis, prevents excessive PAEC proliferation, preserves endothelial barrier function, and has a beneficial effect on animal models of PH.

BMP9 inhibits EC migration and proliferation and usually forms a signaling complex with the TβRII BMPR-II and the TβRI ALK1, which is expressed mainly in ECs. This complex causes the phosphorylation and activation of SMAD1, SMAD5 and SMAD9 David et al. (2007). In human PAEC cultures, BMP9 dose-dependently increases the expression of BMPR2 and ID1, and upregulates SMAD1, SMAD5, and SMAD9 phosphorylation Long et al. (2015). Additionally, in human PAECs BMP9 prevents JNK phosphorylation, caspase-3 cleavage, and apoptosis through BMPR2. Moreover, in rats, BMP9 reversed PH induced by monocrotaline or by a combination of hypoxia and Sugen. Notably, BMP9 prevented the muscularization of small vessels in the lung and the enlargement of the right heart ventricle caused by hypoxia and Sugen Long et al. (2015). Also, in human PAECs, BMP9 can associate with ACVR2A and ALK1 resulting in the phosphorylation of SMAD2 but not SMAD3. The activity of SMAD2 is necessary for the expression of E-selectin and IL8 caused by BMP9 Upton et al. (2009). Further, heterozygous loss of function mutations in the gene GDF2 that encodes BMP9 are likely the cause of PH in some patients Hodgson et al. (2020). Loss of BMPR2 function in human PAECs reverses the beneficial effects of BMP9, in this context BMP9 promotes human PAEC proliferation and increases angiogenesis in mice Theilmann et al. (2020).

2 SUPPLEMENTARY TABLES AND FIGURES

For more information on Supplementary Material and for details on the different file types accepted, please see the Supplementary Material section of the Author Guidelines.

Figures, tables, and images will be published under a Creative Commons CC-BY licence and permission must be obtained for use of copyrighted material from other sources (including re-published/adapted/modified/partial figures and images from the internet). It is the responsibility of the authors to acquire the licenses, to follow any citation instructions requested by third-party rights holders, and cover any supplementary charges.

2.1 Tables

Entry	Edge type
1	activeUnitOf
2	candidateOf
3	catalystOf
4	catalyzes
5	componentOf
6	input
7	memberOf
8	output
9	regulates
10	regulatorOf
11	repeatedUnitOf
12	requiredInput

Table S1. Edge types included in algorithmic analyses performed on the Reactome graph database.

2.2 Figures

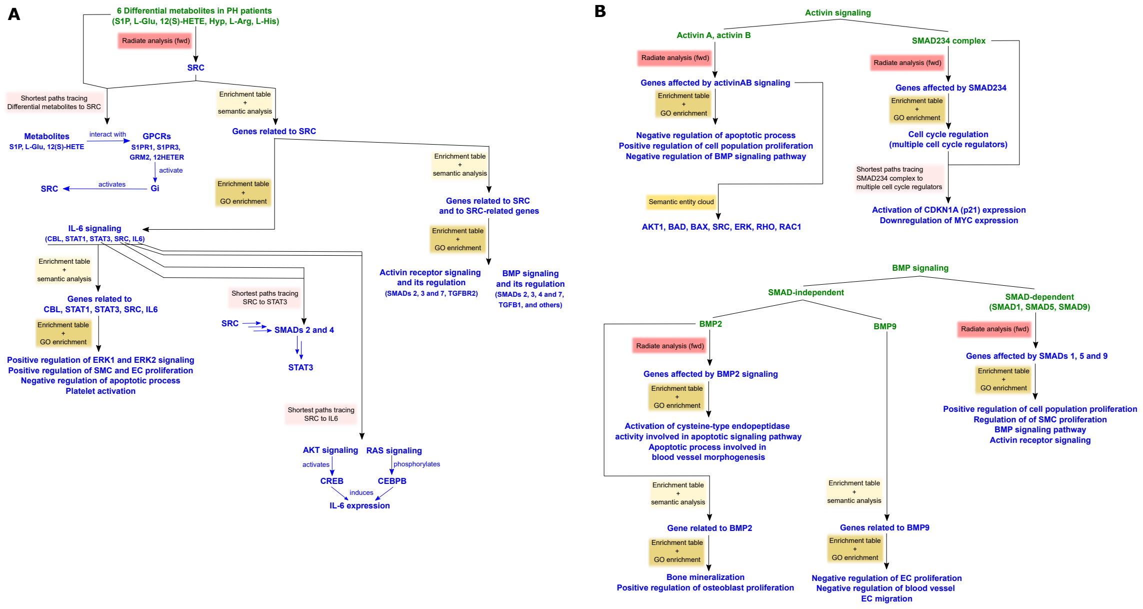

Figure S1. Workflows illustrating the combinatorial application of *Lifelike*'s data analytics methods to identify key biological processess in pulmonary hypertension. The analysis workflow was initiated either with six differential metabolites detected in PH patient plasma (A) and led us to analyze Activin and BMP signaling (B).

REFERENCES

Abou-Jaoude, A., Badiqué, L., Mlih, M., Awan, S., Guo, S., Lemle, A., et al. (2018). Loss of the adaptor protein ShcA in endothelial cells protects against monocyte macrophage adhesion, LDL-oxydation, and atherosclerotic lesion formation. *Scientific reports* 8, 4501. doi:10.1038/s41598-018-22819-3

Alsaffar, H., Martino, N., Garrett, J. P., and Adam, A. P. (2018). Interleukin-6 promotes a sustained loss of endothelial barrier function via Janus kinase-mediated STAT3 phosphorylation and de novo protein synthesis. *American Journal of Physiology-Cell Physiology* 314, C589–C602. doi:10.1152/ajpcell.00235.2017

Amberg, G. C. and Navedo, M. F. (2013). Calcium dynamics in vascular smooth muscle. *Microcirculation* 20, 281–289. doi:10.1111/micc.12046

Anderson, L., Lowery, J. W., Frank, D. B., Novitskaya, T., Jones, M., Mortlock, D. P., et al. (2010). Bmp2 and Bmp4 exert opposing effects in hypoxic pulmonary hypertension. *American Journal of Physiology-Regulatory, Integrative and Comparative Physiology* 298, R833–R842. doi:10.1152/ajpregu.00534.2009

Antonetti, D. A., Barber, A. J., Hollinger, L. A., Wolpert, E. B., and Gardner, T. W. (1999). Vascular endothelial growth factor induces rapid phosphorylation of tight junction proteins occludin and zonula occluden 1: a potential mechanism for vascular permeability in diabetic retinopathy and tumors. *Journal of Biological Chemistry* 274, 23463–23467. doi:10.1074/jbc.274.33.23463

Aykul, S. and Martinez-Hackert, E. (2016). Transforming growth factor-β family ligands can function as antagonists by competing for type II receptor binding. *Journal of Biological Chemistry* 291, 10792–10804. doi:10.1074/jbc.M115.713487

Beets, K., Staring, M. W., Criem, N., Maas, E., Schellinx, N., de Sousa Lopes, S. M. C., et al. (2016). BMP-SMAD signalling output is highly regionalized in cardiovascular and lymphatic endothelial networks. *BMC Developmental Biology* 16, 1–16. doi:10.1186/s12861-016-0133-x

Benn, A., Hiepen, C., Osterland, M., Schütte, C., Zwijsen, A., and Knaus, P. (2017). Role of bone morphogenetic proteins in sprouting angiogenesis: differential BMP receptor-dependent signaling pathways balance stalk vs. tip cell competence. *The FASEB Journal* 31, 4720. doi:10.1096/fj.201700193RR

Birukova, A. A., Shah, A. S., Tian, Y., Gawlak, G., Sarich, N., and Birukov, K. G. (2016). Selective role of vinculin in contractile mechanisms of endothelial permeability. *American Journal of Respiratory Cell and Molecular Biology* 55, 476–486. doi:10.1165/rcmb.2015-0328OC

Cantalupo, A., Gargiulo, A., Dautaj, E., Liu, C., Zhang, Y., Hla, T., et al. (2017). S1PR1 (sphingosine-1-phosphate receptor 1) signaling regulates blood flow and pressure. *Hypertension* 70, 426–434. doi:10.1161/HYPERTENSIONAHA.117.09088

Caricasole, A., Sala, C., Roncarati, R., Formenti, E., and Terstappen, G. C. (2000). Cloning and characterization of the human phosphoinositide-specific phospholipase C-beta 1 (PLCβ1). *Biochimica et Biophysica Acta (BBA)-Gene Structure and Expression* 1517, 63–72. doi:10.1016/S0167-4781(00)00260-8

Chang, A. C., Fu, Y., Garside, V. C., Niessen, K., Chang, L., Fuller, M., et al. (2011). Notch initiates the endothelial-to-mesenchymal transition in the atrioventricular canal through autocrine activation of soluble guanylyl cyclase. *Developmental cell* 21, 288–300. doi:10.1016/j.devcel.2011.06.022

Coggins, M. P. and Bloch, K. D. (2007). Nitric oxide in the pulmonary vasculature. *Arteriosclerosis, thrombosis, and vascular biology* 27, 1877–1885. doi:10.1161/ATVBAHA.107.142943

Conery, A. R., Cao, Y., Thompson, E. A., Townsend Jr, C. M., Ko, T. C., and Luo, K. (2004). Akt interacts directly with Smad3 to regulate the sensitivity to TGF-β-induced apoptosis. *Nature cell biology* 6, 366–372. doi:10.1038/ncb1117

Cullere, X., Shaw, S. K., Andersson, L., Hirahashi, J., Luscinskas, F. W., and Mayadas, T. N. (2005). Regulation of vascular endothelial barrier function by Epac, a cAMP-activated exchange factor for Rap GTPase. *Blood* 105, 1950–1955. doi:10.1182/blood-2004-05-1987

David, L., Mallet, C., Mazerbourg, S., Feige, J.-J., and Bailly, S. (2007). Identification of BMP9 and BMP10 as functional activators of the orphan activin receptor-like kinase 1 (ALK1) in endothelial cells. *Blood* 109, 1953–1961. doi:10.1182/blood-2006-07-034124

de Jesus Perez, V. A., Alastalo, T.-P., Wu, J. C., Axelrod, J. D., Cooke, J. P., Amieva, M., et al. (2009). Bone morphogenetic protein 2 induces pulmonary angiogenesis via Wnt–β-catenin and Wnt–Rhoa–Rac1 pathways. *The Journal of cell biology* 184, 83–99. doi:10.1083/jcb.200806049

de Vinuesa, A. G., Abdelilah-Seyfried, S., Knaus, P., Zwijsen, A., and Bailly, S. (2016). BMP signaling in vascular biology and dysfunction. *Cytokine & growth factor reviews* 27, 65–79. doi:10.1016/j.cytogfr.2015.12.005

Domingues, A. and Fantin, A. (2021). Neuropilin 1 regulation of vascular permeability signaling. *Biomolecules* 11, 666. doi:10.3390/biom11050666

El-Bizri, N., Wang, L., Merklinger, S. L., Guignabert, C., Desai, T., Urashima, T., et al. (2008). Smooth Muscle Protein 22α–Mediated Patchy Deletion of Bmpr1a impairs cardiac contractility but protects against pulmonary vascular remodeling. *Circulation research* 102, 380–388. doi:10.1161/CIRCRESAHA.107.161059

Eulenfeld, R., Dittrich, A., Khouri, C., Müller, P. J., Mütze, B., Wolf, A., et al. (2012). Interleukin-6 signalling: more than jaks and STATs. *European journal of cell biology* 91, 486–495. doi:10.1016/j.ejcb.2011.09.010

Frank, D. B., Lowery, J., Anderson, L., Brink, M., Reese, J., and de Caestecker, M. (2008). Increased susceptibility to hypoxic pulmonary hypertension in Bmpr2 mutant mice is associated with endothelial dysfunction in the pulmonary vasculature. *American Journal of Physiology-Lung Cellular and Molecular Physiology* 294, L98–L109. doi:10.1152/ajplung.00034.2007

Gavard, J. and Gutkind, J. S. (2006). VEGF controls endothelial-cell permeability by promoting the β-arrestin-dependent endocytosis of VE-cadherin. *Nature cell biology* 8, 1223–1234. doi:10.1038/ncb1486

Gill, S. S., Mueller, R. W., Mcguire, P. F., and Pulido, O. M. (2000). Potential target sites in peripheral tissues for excitatory neurotransmission and excitotoxicity. *Toxicologic pathology* 28, 277–284. doi:10.1177/01926233000280

Groppe, J., Hinck, C. S., Samavarchi-Tehrani, P., Zubieta, C., Schuermann, J. P., Taylor, A. B., et al. (2008). Cooperative assembly of TGF-β superfamily signaling complexes is mediated by two disparate mechanisms and distinct modes of receptor binding. *Molecular cell* 29, 157–168. doi:10.1016/j.molcel.2007.11.039

Guignabert, C., Savale, L., Boucly, A., Thuillet, R., Tu, L., Ottaviani, M., et al. (2023). Serum and Pulmonary Expression Profiles of the Activin Signaling System in Pulmonary Arterial Hypertension. *Circulation* 147, 1809–1822. doi:10.1161/CIRCULATIONAHA.122.061501

Guo, Y., Zhang, W., Giroux, C., Cai, Y., Ekambaram, P., Dilly, A.-k., et al. (2011). Identification of the orphan G protein-coupled receptor gpr31 as a receptor for 12-(S)-hydroxyeicosatetraenoic acid. *Journal of Biological Chemistry* 286, 33832–33840. doi:10.1074/jbc.M110.216564

Hardie, W. D., Korfhagen, T. R., Sartor, M. A., Prestridge, A., Medvedovic, M., Le Cras, T. D., et al. (2007). Genomic profile of matrix and vasculature remodeling in TGF-α–induced pulmonary fibrosis. *American Journal of Respiratory Cell and Molecular Biology* 37, 309–321. doi:10.1165/rcmb.2006-0455OC

Hashimoto-Kataoka, T., Hosen, N., Sonobe, T., Arita, Y., Yasui, T., Masaki, T., et al. (2015). Interleukin-6/interleukin-21 signaling axis is critical in the pathogenesis of pulmonary arterial hypertension. *Proceedings of the National Academy of Sciences* 112, E2677–E2686. doi:10.1073/pnas.142477411

Hata, A. and Chen, Y.-G. (2016). TGF-β signaling from receptors to smads. *Cold Spring Harbor perspectives in biology* 8, a022061. doi:10.1101/cshperspect.a022061

Heinecke, K., Seher, A., Schmitz, W., Mueller, T. D., Sebald, W., and Nickel, J. (2009). Receptor oligomerization and beyond: a case study in bone morphogenetic proteins. *BMC biology* 7, 1–20. doi:10.1186/1741-7007-7-59

Hirano, T., Ishihara, K., and Hibi, M. (2000). Roles of STAT3 in mediating the cell growth, differentiation and survival signals relayed through the il-6 family of cytokine receptors. *Oncogene* 19, 2548–2556. doi:10.1038/sj.onc.1203551

Hodgson, J., Swietlik, E. M., Salmon, R. M., Hadinnapola, C., Nikolic, I., Wharton, J., et al. (2020). Characterization of GDF2 mutations and levels of BMP9 and BMP10 in pulmonary arterial hypertension. *American Journal of Respiratory and Critical Care Medicine* 201, 575–585. doi:10.1164/rccm.201906-1141OC

Huang, Y.-H., Yang, H.-Y., Hsu, Y.-F., Chiu, P.-T., Ou, G., and Hsu, M.-J. (2014). Src contributes to IL6-induced vascular endothelial growth factor-C expression in lymphatic endothelial cells. *Angiogenesis* 17, 407–418. doi:10.1007/s10456-013-9386-1

Jain, R., Watson, U., Vasudevan, L., and Saini, D. K. (2018). ERK activation pathways downstream of GPCRs. *International review of cell and molecular biology* 338, 79–109. doi:10.1016/bs.ircmb.2018.02.003

Jiang, L., Qi, Y., Kong, X., Wang, R., Qi, J., Lin, F., et al. (2021). Activin A as a novel chemokine induces migration of L929 fibroblasts by ERK signaling in microfluidic devices. *Frontiers in Cell and Developmental Biology* 9, 660316. doi:10.3389/fcell.2021.660316

Julio-Pieper, M., Flor, P. J., Dinan, T. G., and Cryan, J. F. (2011). Exciting times beyond the brain: metabotropic glutamate receptors in peripheral and non-neural tissues. *Pharmacological reviews* 63, 35–58. doi:10.1124/pr.110.004036

Jung, J. E., Lee, H.-G., Cho, I.-H., Chung, D. H., Yoon, S.-H., Yang, Y. M., et al. (2005). STAT3 is a potential modulator of HIF-1-mediated VEGF expression in human renal carcinoma cells. *The FASEB Journal* 19, 1296–1298. doi:10.1096/fj.04-3099fje

Kaneda, H., Arao, T., Matsumoto, K., De Velasco, M., Tamura, D., Aomatsu, K., et al. (2011). Activin A inhibits vascular endothelial cell growth and suppresses tumour angiogenesis in gastric cancer. *British journal of cancer* 105, 1210–1217. doi:10.1038/bjc.2011.348

Kevil, C. G., Payne, D. K., Mire, E., and Alexander, J. S. (1998). Vascular permeability factor/vascular endothelial cell growth factor-mediated permeability occurs through disorganization of endothelial junctional proteins. *Journal of Biological Chemistry* 273, 15099–15103. doi:10.1074/jbc.273.24.15099

Klein, E. A. and Assoian, R. K. (2008). Transcriptional regulation of the cyclin D1 gene at a glance. *Journal of cell science* 121, 3853–3857. doi:10.1242/jcs.039131

Kokabu, S., Nojima, J., Kanomata, K., Ohte, S., Yoda, T., Fukuda, T., et al. (2010). Protein phosphatase magnesium-dependent 1A–mediated inhibition of bmp signaling is independent of smad dephosphorylation. *Journal of bone and mineral research* 25, 653–660. doi:10.1359/jbmr.090736

Kolliputi, N. and Waxman, A. B. (2009). IL-6 cytoprotection in hyperoxic acute lung injury occurs via PI3K/Akt-mediated Bax phosphorylation. *American Journal of Physiology-Lung Cellular and Molecular Physiology* 297, L6–L16. doi:10.1152/ajplung.90381.2008

Kosaki, K., Kosaki, R., Bassi, M., Lewin, M., Belmont, J., Schauer, G., et al. (1999). Characterization and mutation analysis of human LEFTY a and LEFTY b, homologues of murine genes implicated in left-right axis development. *The American Journal of Human Genetics* 64, 712–721. doi:10.1086/302289

Kulikauskas, M. R. and Bautch, V. L. (2022). The versatility and paradox of BMP signaling in endothelial cell behaviors and blood vessel function. *Cellular and Molecular Life Sciences* 79, 77. doi:10.1007/s00018-021-04033-z

Lavoie, H., Gagnon, J., and Therrien, M. (2020). ERK signalling: a master regulator of cell behaviour, life and fate. *Nature reviews Molecular cell biology* 21, 607–632. doi:10.1038/s41580-020-0255-7

Li, Q., Li, Y., Lei, C., Tan, Y., and Yi, G. (2021). Sphingosine-1-phosphate receptor 3 signaling. *Clinica Chimica Acta* 519, 32–39. doi:10.1016/j.cca.2021.03.025

Lincoln, T. M., Dey, N., and Sellak, H. (2001). Invited review: cGMP-dependent protein kinase signaling mechanisms in smooth muscle: from the regulation of tone to gene expression. *Journal of applied physiology* 91, 1421–1430. doi:10.1152/jappl.2001.91.3.1421

Long, L., Ormiston, M. L., Yang, X., Southwood, M., Gräf, S., Machado, R. D., et al. (2015). Selective enhancement of endothelial BMPR-II with BMP9 reverses pulmonary arterial hypertension. *Nature medicine* 21, 777–785. doi:10.1038/nm.3877

Lowery, J. W., Frump, A. L., Anderson, L., DiCarlo, G. E., Jones, M. T., and de Caestecker, M. P. (2010). ID family protein expression and regulation in hypoxic pulmonary hypertension. *American Journal of Physiology-Regulatory, Integrative and Comparative Physiology* 299, R1463–R1477. doi:10.1152/ajpregu.00866.2009

Lucas, K. A., Pitari, G. M., Kazerounian, S., Ruiz-Stewart, I., Park, J., Schulz, S., et al. (2000). Guanylyl cyclases and signaling by cyclic GMP. *Pharmacological Reviews* 52, 375–414

Makino, Y., Yoon, J.-H., Bae, E., Kato, M., Miyazawa, K., Ohira, T., et al. (2017). Repression of Smad3 by Stat3 and c-Ski/SnoN induces gefitinib resistance in lung adenocarcinoma. *Biochemical and biophysical research communications* 484, 269–277. doi:10.1016/j.bbrc.2017.01.093

Miyazawa, K. and Miyazono, K. (2017). Regulation of TGF-β family signaling by inhibitory Smads. *Cold Spring Harbor perspectives in biology* 9, a022095. doi:10.1101/cshperspect.a022095

Murakami, A., Takasugi, H., Ohnuma, S., Koide, Y., Sakurai, A., Takeda, S., et al. (2010). Sphingosine 1-phosphate (S1P) regulates vascular contraction via S1P3 receptor: investigation based on a new s1p3 receptor antagonist. *Molecular pharmacology* 77, 704–713. doi:10.1124/mol.109.061481

Natarajan, V., Jayaram, H. N., Scribner, W. M., and Garcia, J. (1994). Activation of endothelial cell phospholipase d by sphingosine and sphingosine-1-phosphate. *American Journal of Respiratory Cell and Molecular Biology* 11, 221–229. doi:10.1165/ajrcmb.11.2.8049083

Nguyen, C. H., Stadler, S., Brenner, S., Huttary, N., Krieger, S., Jäger, W., et al. (2016). Cancer cell-derived 12 (S)-HETE signals via 12-HETE receptor, RHO, ROCK and MLC2 to induce lymph endothelial barrier breaching. *British Journal of Cancer* 115, 364–370. doi:10.1038/bjc.2016.201

Papapetropoulos, A., Fulton, D., Mahboubi, K., Kalb, R. G., O'Connor, D. S., Li, F., et al. (2000). Angiopoietin-1 inhibits endothelial cell apoptosis via the Akt/survivin pathway. *Journal of Biological Chemistry* 275, 9102–9105. doi:10.1074/jbc.275.13.9102

Preston, I. R., Hill, N. S., Warburton, R. R., and Fanburg, B. L. (2006). Role of 12-lipoxygenase in hypoxia-induced rat pulmonary artery smooth muscle cell proliferation. *American Journal of Physiology-Lung Cellular and Molecular Physiology* 290, L367–L374. doi:10.1152/ajplung.00114.2005

Pullamsetti, S. S., Seeger, W., Savai, R., et al. (2018). Classical IL-6 signaling: a promising therapeutic target for pulmonary arterial hypertension. *The Journal of Clinical Investigation* 128, 1720–1723. doi:10.1172/JCI120415

Pyne, N. J. and Pyne, S. (2017). Sphingosine 1-phosphate receptor 1 signaling in mammalian cells. *Molecules* 22, 344. doi:10.3390/molecules22030344

Ranchoux, B., Antigny, F., Rucker-Martin, C., Hautefort, A., Péchoux, C., Bogaard, H. J., et al. (2015). Endothelial-to-mesenchymal transition in pulmonary hypertension. *Circulation* 131, 1006–1018. doi:10.1161/CIRCULATIONAHA.114.008750

Ryanto, G. R., Ikeda, K., Miyagawa, K., Tu, L., Guignabert, C., Humbert, M., et al. (2021). An endothelial activin A-bone morphogenetic protein receptor type 2 link is overdriven in pulmonary hypertension. *Nature communications* 12, 1720. doi:10.1038/s41467-021-21961-3

Samitas, K., Tousa, S., Poulos, N., Zervas, E., Semitekolou, M., Hawrylowicz, C. M., et al. (2013). Activin-A is up-regulated in severe asthma, attenuates allergic responses and is associated with angiogenesis. *Clinical and Translational Allergy* 3, 1–1. doi:10.1186/2045-7022-3-S1-O4

Simpson, C. E., Chen, J. Y., Damico, R. L., Hassoun, P. M., Martin, L. J., Yang, J., et al. (2020). Cellular sources of interleukin-6 and associations with clinical phenotypes and outcomes in pulmonary arterial hypertension. *European Respiratory Journal* 55. doi:10.1183/13993003.01761-2019

Singer, C. A., Lontay, B., Unruh, H., Halayko, A. J., and Gerthoffer, W. T. (2011). Src mediates cytokine-stimulated gene expression in airway myocytes through ERK MAPK. *Cell Communication and Signaling* 9, 1–5. doi:10.1186/1478-811X-9-14

Somlyo, A. P. and Somlyo, A. V. (2000). Signal transduction by G-proteins, rho-kinase and protein phosphatase to smooth muscle and non-muscle myosin II. *The Journal of physiology* 522, 177–185. doi:10.1111/j.1469-7793.2000.t01-2-00177.x

Somlyo, A. P. and Somlyo, A. V. (2003). Ca2+ sensitivity of smooth muscle and nonmuscle myosin II: modulated by G proteins, kinases, and myosin phosphatase. *Physiological reviews* 83, 1325–1358. doi:10.1152/physrev.00023.2003

Steiner, M. K., Syrkina, O. L., Kolliputi, N., Mark, E. J., Hales, C. A., and Waxman, A. B. (2009). Interleukin-6 overexpression induces pulmonary hypertension. *Circulation research* 104, 236–244. doi:10.1161/CIRCRESAHA.108.182014

Szilágyi, S. S., Amsalem-Zafran, A. R., Shapira, K. E., Ehrlich, M., and Henis, Y. I. (2022). Competition between type i activin and BMP receptors for binding to ACVR2A regulates signaling to distinct Smad pathways. *BMC biology* 20, 1–21. doi:s12915-022-01252-z

Taha, T. A., Argraves, K. M., and Obeid, L. M. (2004). Sphingosine-1-phosphate receptors: receptor specificity versus functional redundancy. *Biochimica et Biophysica Acta (BBA)-Molecular and Cell Biology of Lipids* 1682, 48–55. doi:10.1016/j.bbalip.2004.01.006

Tang, D. G., Timar, J., Grossi, I. M., Renaud, C., Kimler, V. A., Diglio, C. A., et al. (1993). The lipoxygenase metabolite, 12 (S)-HETE, induces a protein kinase c-dependent cytoskeletal rearrangement and retraction of microvascular endothelial cells. *Experimental cell research* 207, 361–375. doi:10.1006/excr.1993.1203

Ten Dijke, P., Yamashita, H., Ichijo, H., Franzén, P., Laiho, M., Miyazono, K., et al. (1994). Characterization of type I receptors for transforming growth factor-β and activin. *Science* 264, 101–104. doi:10.1126/science.8140412

Theilmann, A. L., Hawke, L. G., Hilton, L. R., Whitford, M. K., Cole, D. V., Mackeil, J. L., et al. (2020). Endothelial BMPR2 loss drives a proliferative response to BMP (bone morphogenetic protein) 9 via prolonged canonical signaling. *Arteriosclerosis, Thrombosis, and Vascular Biology* 40, 2605–2618. doi:10.1161/ATVBAHA.119.313357

Toxvig, A. K., Wehland, M., Grimm, D., Infanger, M., and Krüger, M. (2019). A focus on riociguat in the treatment of pulmonary arterial hypertension. *Basic & clinical pharmacology & toxicology* 125, 202–214. doi:10.1111/bcpt.13272

Trepanier, C., Lei, G., Xie, Y.-F., and MacDonald, J. F. (2013). Group II metabotropic glutamate receptors modify N-methyl-D-aspartate receptors via Src kinase. *Scientific reports* 3, 926. doi:10.1038/srep00926

Tsutsumi, T., Nagaoka, T., Yoshida, T., Wang, L., Kuriyama, S., Suzuki, Y., et al. (2019). Nintedanib ameliorates experimental pulmonary arterial hypertension via inhibition of endothelial mesenchymal

transition and smooth muscle cell proliferation. *PLoS One* 14, e0214697. doi:10.1371/journal.pone.0214697

Tzavlaki, K. and Moustakas, A. (2020). Tgf-β signaling. *Biomolecules* 10, 487. doi:10.3390/biom10030487

Upton, P. D., Davies, R. J., Trembath, R. C., and Morrell, N. W. (2009). Bone morphogenetic protein (BMP) and activin type II receptors balance BMP9 signals mediated by activin receptor-like kinase-1 in human pulmonary artery endothelial cells. *Journal of Biological Chemistry* 284, 15794–15804. doi:10.1074/jbc.M109.002881

Valderrama-Carvajal, H., Cocolakis, E., Lacerte, A., Lee, E.-H., Krystal, G., Ali, S., et al. (2002). Activin/TGF-β induce apoptosis through Smad-dependent expression of the lipid phosphatase SHIP. *Nature cell biology* 4, 963–969. doi:10.1038/ncb885

Vergadi, E., Chang, M. S., Lee, C., Liang, O. D., Liu, X., Fernandez-Gonzalez, A., et al. (2011). Early macrophage recruitment and alternative activation are critical for the later development of hypoxia-induced pulmonary hypertension. *Circulation* 123, 1986–1995. doi:10.1161/CIRCULATIONAHA.110.978627

Verin, A. D., Gilbert-McClain, L. I., Patterson, C. E., and Garcia, J. G. (1998). Biochemical regulation of the nonmuscle myosin light chain kinase isoform in bovine endothelium. *American journal of respiratory cell and molecular biology* 19, 767–776. doi:10.1165/ajrcmb.19.5.3126

von Gersdorff, G., Susztak, K., Rezvani, F., Bitzer, M., Liang, D., and Böttinger, E. P. (2000). Smad3 and Smad4 mediate transcriptional activation of the human Smad7 promoter by transforming growth factor β. *Journal of Biological Chemistry* 275, 11320–11326. doi:10.1074/jbc.275.15.11320

Wallez, Y. and Huber, P. (2008). Endothelial adherens and tight junctions in vascular homeostasis, inflammation and angiogenesis. *Biochimica et Biophysica Acta (BBA)-Biomembranes* 1778, 794–809. doi:10.1016/j.bbamem.2007.09.003

Wang, J., Feng, W., Li, F., Shi, W., Zhai, C., Li, S., et al. (2018). Sphk1/S1P mediates TGF-β1-induced proliferation of pulmonary artery smooth muscle cells and its potential mechanisms. *Pulmonary circulation* 9, 2045894018816977. doi:10.1177/2045894018816977

Watanabe, D., Takagi, H., Suzuma, K., Suzuma, I., Oh, H., Ohashi, H., et al. (2004). Transcription factor Ets-1 mediates ischemia-and vascular endothelial growth factor-dependent retinal neovascularization. *The American Journal of Pathology* 164, 1827–1835. doi:10.1016/S0002-9440(10)63741-8

Watterson, K. R., Ratz, P. H., and Spiegel, S. (2005). The role of sphingosine-1-phosphate in smooth muscle contraction. *Cellular signalling* 17, 289–298. doi:10.1016/j.cellsig.2004.09.013

Wilkerson, B. A. and Argraves, K. M. (2014). The role of sphingosine-1-phosphate in endothelial barrier function. *Biochimica et Biophysica Acta (BBA)-Molecular and Cell Biology of Lipids* 1841, 1403–1412. doi:10.1016/j.bbalip.2014.06.012

Wong, W. K., Knowles, J. A., and Morse, J. H. (2005). Bone morphogenetic protein receptor type II C-terminus interacts with c-Src: implication for a role in pulmonary arterial hypertension. *American Journal of Respiratory Cell and Molecular Biology* 33, 438–446. doi:10.1165/rcmb.2005-0103OC

Wu, J., Yu, Z., and Su, D. (2014). BMP4 protects rat pulmonary arterial smooth muscle cells from apoptosis by PI3K/AKT/Smad1/5/8 signaling. *International Journal of Molecular Sciences* 15, 13738–13754. doi:10.3390/ijms150813738

Yagi, K., Furuhashi, M., Aoki, H., Goto, D., Kuwano, H., Sugamura, K., et al. (2002). c-myc Is a Downstream Target of the Smad Pathway* 210. *Journal of Biological Chemistry* 277, 854–861. doi:10.1074/jbc.M104170200

Yan, X., Lin, Z., Chen, F., Zhao, X., Chen, H., Ning, Y., et al. (2009). Human BAMBI cooperates with Smad7 to inhibit transforming growth factor-β signaling. *Journal of Biological Chemistry* 284, 30097–30104. doi:10.1074/jbc.M109.049304

Yang, P., Troncone, L., Augur, Z. M., Kim, S. S., McNeil, M. E., and Paul, B. Y. (2020). The role of bone morphogenetic protein signaling in vascular calcification. *Bone* 141, 115542. doi:10.1016/j.bone.2020.115542

Yao, X., Kwan, H., Chan, F., Chan, N., and Huang, Y. (2000). A protein kinase g-sensitive channel mediates flow-induced ca2+ entry into vascular endothelial cells. *The FASEB Journal* 14, 932–938. doi:10.1096/fasebj.14.7.932

Ye, D., Liu, Y., Pan, H., Feng, Y., Lu, X., Gan, L., et al. (2023). Insights into bone morphogenetic proteins in cardiovascular diseases. *Frontiers in Pharmacology* 14, 1125642. doi:10.3389/fphar.2023.1125642

Yu, G., Shen, P., Lee, Y.-C., Pan, J., Song, J. H., Pan, T., et al. (2021). Multiple pathways coordinating reprogramming of endothelial cells into osteoblasts by BMP4. *iScience* 24. doi:10.1016/j.isci.2021.102388

Yu, J., Pan, L., Qin, X., Chen, H., Xu, Y., Chen, Y., et al. (2010). MTMR4 attenuates transforming growth factor β (TGFβ) signaling by dephosphorylating R-smads in endosomes. *Journal of Biological Chemistry* 285, 8454–8462. doi:10.1074/jbc.M109.075036

Yun, J.-H., Park, S. W., Kim, K.-J., Bae, J.-S., Lee, E. H., Paek, S. H., et al. (2017). Endothelial STAT3 activation increases vascular leakage through downregulating tight junction proteins: implications for diabetic retinopathy. *Journal of cellular physiology* 232, 1123–1134. doi:10.1002/jcp.25575

Zegeye, M. M., Lindkvist, M., Fälker, K., Kumawat, A. K., Paramel, G., Grenegård, M., et al. (2018). Activation of the JAK/STAT3 and PI3K/AKT pathways are crucial for IL-6 trans-signaling-mediated pro-inflammatory response in human vascular endothelial cells. *Cell Communication and Signaling* 16, 1–10. doi:10.1186/s12964-018-0268-4

Zhang, C., Lv, J., He, Q., Wang, S., Gao, Y., Meng, A., et al. (2014a). Inhibition of endothelial ERK signalling by Smad1/5 is essential for haematopoietic stem cell emergence. *Nature communications* 5, 3431. doi:10.1038/ncomms443

Zhang, L., Xu, P., Wang, X., Zhang, M., Yan, Y., Chen, Y., et al. (2017). Activin B regulates adipose-derived mesenchymal stem cells to promote skin wound healing via activation of the MAPK signaling pathway. *The international journal of biochemistry & cell biology* 87, 69–76. doi:10.1016/j.biocel.2017.04.004

Zhang, M., Sun, L., Wang, X., Chen, S., Kong, Y., Liu, N., et al. (2014b). Activin B promotes BMSC-mediated cutaneous wound healing by regulating cell migration via the JNK—ERK signaling pathway. *Cell transplantation* 23, 1061–1073. doi:10.3727/096368913X666

Zhang, R.-G., Niu, Y., Pan, K.-W., Pang, H., Chen, C.-L., Yip, C.-Y., et al. (2021). β 2-Adrenoceptor Activation Stimulates IL-6 production via PKA, ERK1/2, Src, and Beta-Arrestin2 signaling pathways in human bronchial epithelia. *Lung* 199, 619–627. doi:10.1007/s00408-021-00484-0

Zhang, Y. E. (2009). Non-Smad pathways in TGF-β signaling. *Cell research* 19, 128–139. doi:10.1038/cr.2008.328
