# Supplementary material for: A Lifelike guided journey through the pathophysiology of pulmonary hypertension—from measured metabolites to the mechanism of action of drugs
Source: Front Cardiovasc Med. 2024 May 23;11:1341145. doi: 10.3389/fcvm.2024.1341145 (PMC11153715; doi:10.3389/fcvm.2024.1341145)
Supplement: Supplementary file 3 [file Presentation3.pdf]

1) Radiate analysis with the upregulated and downregulated metabolites as sources

Radiate_analysis_for_s1p_glu_12hete_hyp_arg_hist.xlsx

R-ALL-83957	Hyp [extracellular region]
R-ALL-351977	L-Arg [extracellular region]
R-ALL-210382	L-Glu [extracellular region]
R-ALL-419305	S1P [extracellular region]
R-ALL-3296298	12(S)-HETE [extracellular region]
R-ALL-351970	L-His [extracellular region]

Sources

stId	displayName	pagerank
R-HSA-380091	Ligand:GPCR complexes that activate Gi [plasma membrane]	0,042766791
R-ALL-179747	ligands of SLC29A2 [extracellular region]	0,039404389
R-HSA-109527	Equilibrative transport (import) of nucleosides and free bases by solute carrie	0,031465133
R-ALL-179742	ligands of SLC29A2 [cytosol]	0,029183131
R-HSA-3499321	GPR31:12(S)-HETE [plasma membrane]	0,028349993
R-HSA-749445	Ligand:GPCR complexes that activate Gi:Heterotrimeric G-protein Gi (active) [	0,027604233
R-HSA-109529	Equilibrative transport (export) of nucleosides and free bases by solute carrie	0,024589483
R-HSA-749454	The Ligand:GPCR:Gi complex dissociates	0,023472061
R-HSA-749455	Ligand:GPCR complexes that activate Gi:Heterotrimeric G-protein Gi (inactive)	0,022660246
R-HSA-380073	Liganded Gi-activating GPCR acts as a GEF for Gi	0,01782534
R-HSA-109527	Equilibrative transport (import) of nucleosides and free bases by solute carrie	0,016225859
R-HSA-3296304	GPR31 is a receptor for 12(S)-HETE	0,015324623
R-ALL-113599	Hyp [cytosol]	0,014646529
R-HSA-749456	Liganded Gi-activating GPCRs bind inactive heterotrimeric G-protein Gi	0,012247748
R-HSA-9690450	S1PR1:S1P [plasma membrane]	0,011339998
R-HSA-419346	S1PR2-5:S1P [plasma membrane]	0,011339998
	guanyl-nucleotide exchange factor activity of Ligand:GPCR complexes that act	0,009633262
R-ALL-375468	SLC6A14 ligands [extracellular region]	0,009556406
R-HSA-167434	G-protein beta-gamma complex [plasma membrane]	0,007987795
R-HSA-392161	G alpha (i): GTP [plasma membrane]	0,007553266

The most significant targets include many components of G protein signaling pathways

Forward_traces_for_s1p_glu_12hete_hyp_arg_hist.graph

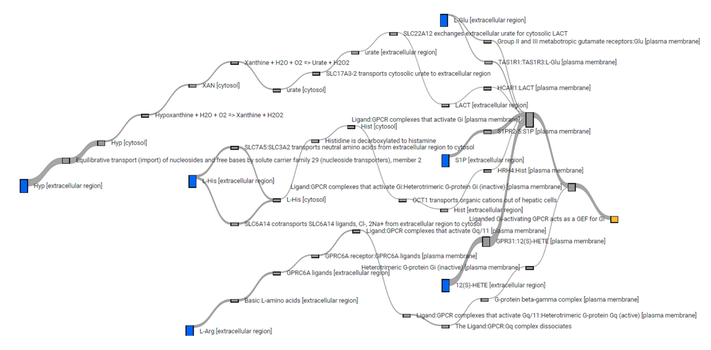

S1P, 12(s)hete, and L-Glu activate Gi signaling

2) SRC is a convergence point for S1P, 12(s)HETE ,and L-Glu

stId	displayName	geneName	pagerank
R-HSA-8964276	p-Y416-SRC [cytosol]	SRC	0,000928258
R-HSA-8964323	Active BTK [cytosol]	BTK	0,000172967
R-HSA-391959	P2RY12 [plasma membrane]	P2RY12	0,000127777
R-HSA-195017	ARHGEF6 [cytosol]	ARHGEF6	6,77634E-05
R-HSA-8964622	Active PAK1 [cytosol]	PAK1	6,77634E-05
R-HSA-391837	TBXA2R [plasma membrane]	TBXA2R	4,71603E-05
R-HSA-418898	ADORA2B [plasma membrane]	ADORA2B	2,6797E-05
R-HSA-391956	P2RY1 [plasma membrane]	P2RY1	2,39873E-05
R-HSA-391834	PTGIR [plasma membrane]	PTGIR	2,3563E-05
R-HSA-167679	p-4Y-PLCG1 [plasma membrane]	PLCG1	1,83755E-05
R-HSA-428935	p-T180,Y182-MAPK14 [cytosol]	MAPK14	1,48092E-05
R-HSA-194371	p-T,Y-MAPK8 [cytosol]	MAPK8	1,02316E-05
R-HSA-399816	p-T508-LIMK1 [cytosol]	LIMK1	8,80459E-06
R-HSA-186833	RASA1 [cytosol]	RASA1	7,66306E-06
R-HSA-162629	PAK1 [cytosol]	PAK1	7,64617E-06
R-HSA-210514	STXBP1-1 [cytosol]	STXBP1	7,12242E-06
R-HSA-168786	SYT1 [plasma membrane]	SYT1	7,12242E-06
R-HSA-210461	RAB3A [plasma membrane]	RAB3A	7,12242E-06
R-HSA-210385	CPLX1 [cytosol]	CPLX1	7,12242E-06
R-HSA-195131	SRGAP2 [cytosol]	SRGAP2	6,87986E-06
R-HSA-1234131	2xHP-HIF1A [cytosol]	HIF1A	5,95927E-06
R-HSA-1234149	2xHP-EPAS1 [cytosol]	EPAS1	5,95927E-06

The non-receptor tyrosine knases SRC and BTK are the most significant protein targets of the metabolites

Watterson, K. R., Ratz, P. H. & Spiegel, S. The role of sphingosine-1-phosphate in smooth muscle contraction. Cell Signal 17, 289–298 (2005).

Murakami, A. et al. Sphingosine 1-Phosphate (S1P) Regulates Vascular Contraction via S1P3 Receptor: Investigation Based on a New S1P3 Receptor Antagonist. Mol Pharmacol 77, 704–713 (2010).

Tang, D. G. et al. The Lipoxygenase Metabolite, 12(S)-HETE, Induces a Protein Kinase C-Dependent Cytoskeletal Rearrangement and Retraction of Microvascular Endothelial Cells. Exp Cell Res 207, 361–375 (1993).

Liu, P. et al. Inhibition of Src activation reverses pulmonary vascular remodeling in experimental pulmonary arterial hypertension via Akt/mTOR/HIF-1<alpha> signaling pathway. Exp Cell Res 380, 36–46 (2019).

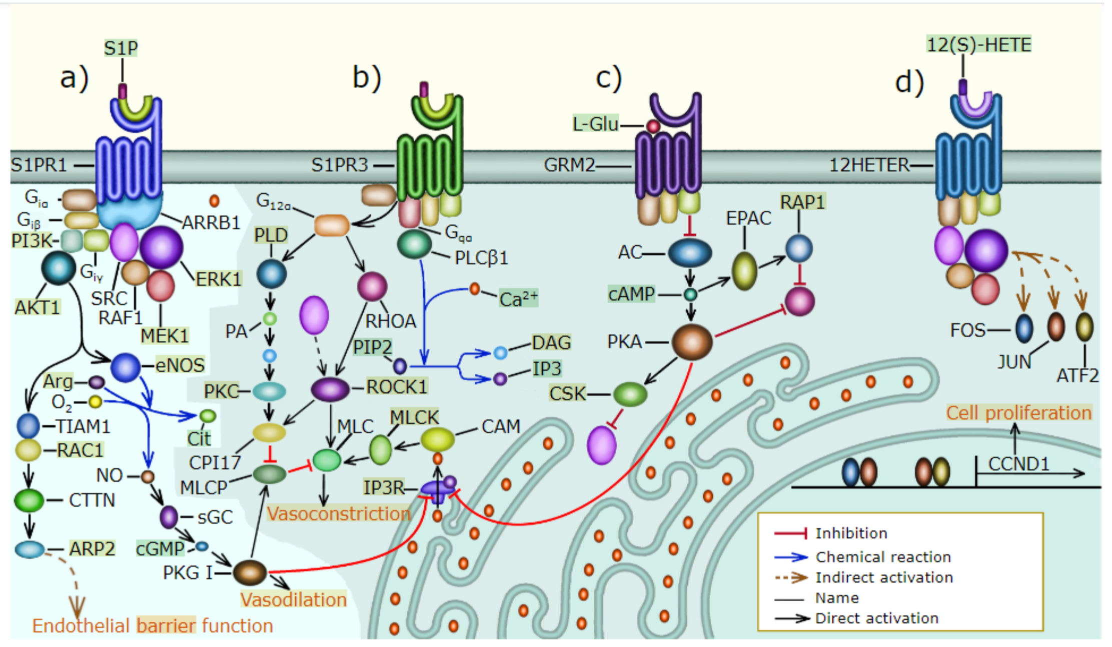

a) S1PR1 signaling, b) S1PR3 signaling, c) L-Glu signaling, d) 12(S)HETE signaling

3) Biological mechanisms associated with SRC

Semantic analysis of SRC

Biological Process			
GOID	GO Term	Associated genes	q-value
70102	interleukin-6-mediated signaling pathway	STAT3, IL6, SRC, CBL, STAT1	6.76e-7 ***
42127	regulation of cell population proliferation	STAT3, TNK2, SRC, JUP, STAT1	5.04e-3**
3340	negative regulation of mesenchymal to epithelial transition involved in metanephros morphogenesis	STAT1, CTNNB1	5.04e-3**
2000635	negative regulation of primary miRNA processing	IL6, STAT3	5.04e-3**
48041	focal adhesion assembly	PDPK1, CTTN, SRC	6.40e-3**

Biological process associated with SRC thorough statistical enrichment

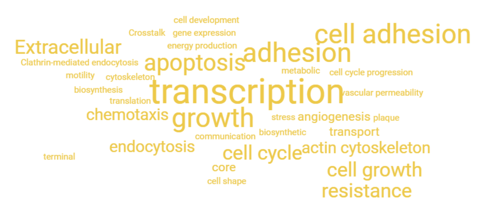

Phenotypes associated with SRC (Count per row word cloud)

4) How is SRC associated with IL6 signaling?

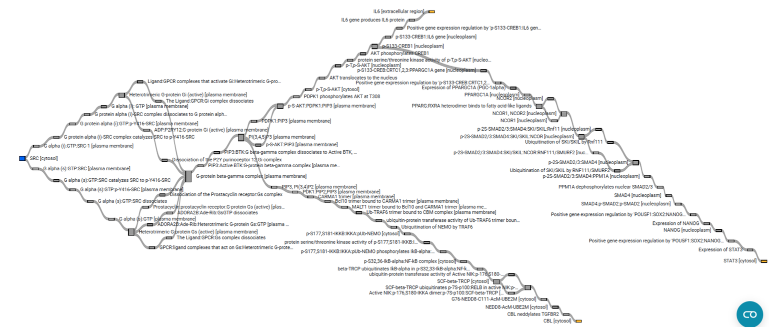

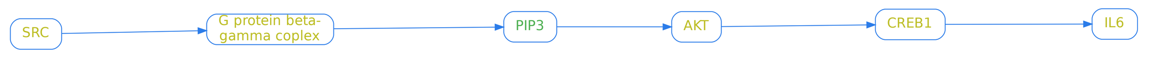

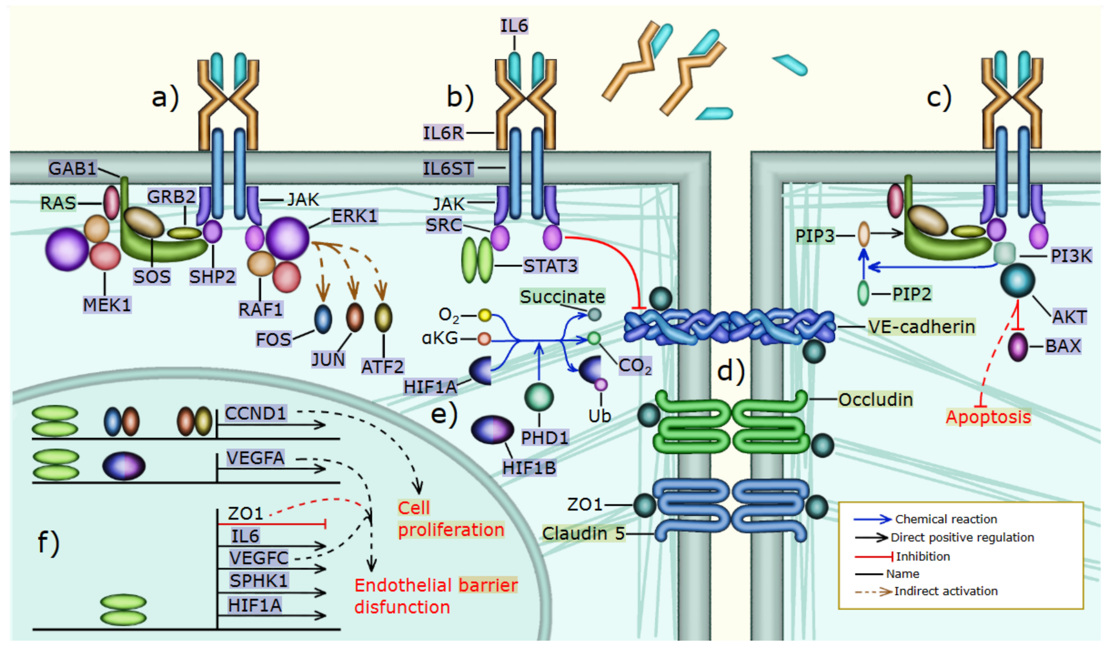

Singer, C. A., Lontay, B., Unruh, H., Halayko, A. J. & Gerthoffer, W. T. Src mediates cytokine-stimulated gene expression in airway myocytes through ERK MAPK. Cell Communication and Signaling 9, 1–5 (2011).

Simpson, C. E. et al. Cellular sources of interleukin-6 and associations with clinical phenotypes and outcomes in pulmonary arterial hypertension. European Respiratory Journal 55, (2020).

Steiner, M. K. et al. IL-6 Overexpression Induces Pulmonary Hypertension. Circ Res 104, 236 (2009).

Hirano, T., Ishihara, K. & Hibi, M. Roles of STAT3 in mediating the cell growth, differentiation and survival signals relayed through the IL-6 family of cytokine receptors. Oncogene 2000 19:21 19, 2548–2556 (2000).

Alsaffar, H., Martino, N., Garrett, J. P. & Adam, A. P. Interleukin-6 promotes a sustained loss of endothelial barrier function via janus kinase-mediated STAT3 phosphorylation and de novo protein synthesis. Am J Physiol Cell Physiol 314, C589–C602 (2020).

Kolliputi, N. & Waxman, A. B. IL-6 cytoprotection in hyperoxic acute lung injury occurs via PI3K/Akt-mediated Bax phosphorylation. Am J Physiol Lung Cell Mol Physiol 297, 6–16 (2009).

5) SRC and IL6 signaling is associated with Activin and BMP signaling

Tielemans, B., Delcroix, M., Belge, C. & Quarck, R. TGFβ and BMPRII signalling pathways in the pathogenesis of pulmonary arterial hypertension. Drug Discov Today 24, 703–716 (2019).

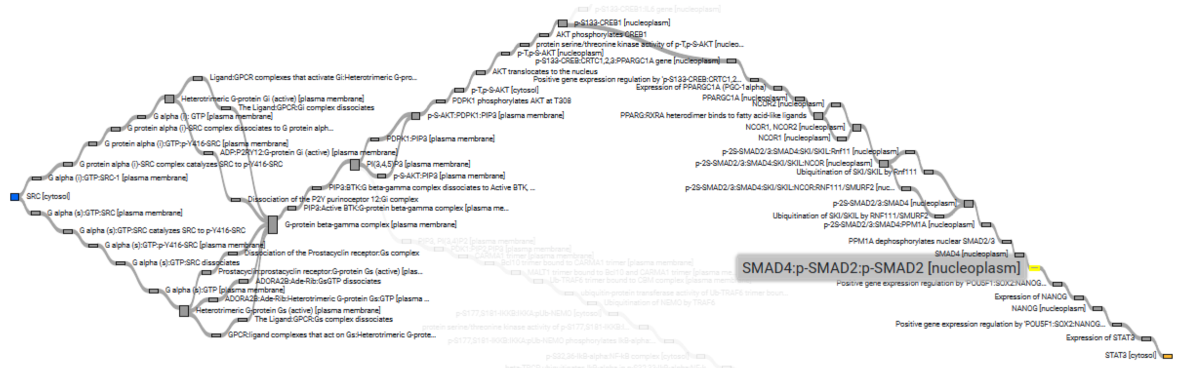

SMAD2 is present in the shortest path that connects SRC to STAT3

Twice expanded semantic analysis around SRCSRC x2

Biological Process		
Search: Activin		
GOID	GO Term	Associated genes
32924	activin receptor signaling pathway	SMAD2, SMAD3, TGFBR2
32925	regulation of activin receptor signaling pathway	SMAD7

Biological Process		
Search: BMP		
GOID	GO Term	Associated genes
30509	BMP signaling pathway	SMAD4, SMAD2, RUNX2, LEF1, SMAD3, TGFB1, ENG, SMAD7, TGFBR3
30513	positive regulation of BMP signaling pathway	SMAD4, SMAD2, FBXL15, ENG, NOTCH1
30514	negative regulation of BMP signaling pathway	ABL1, SMAD4, PPARG, NOTCH1, SMAD7
71773	cellular response to BMP stimulus	RUNX2, SOX9, SMAD4

6) Activin and BMP signaling in pulmonary hypertension

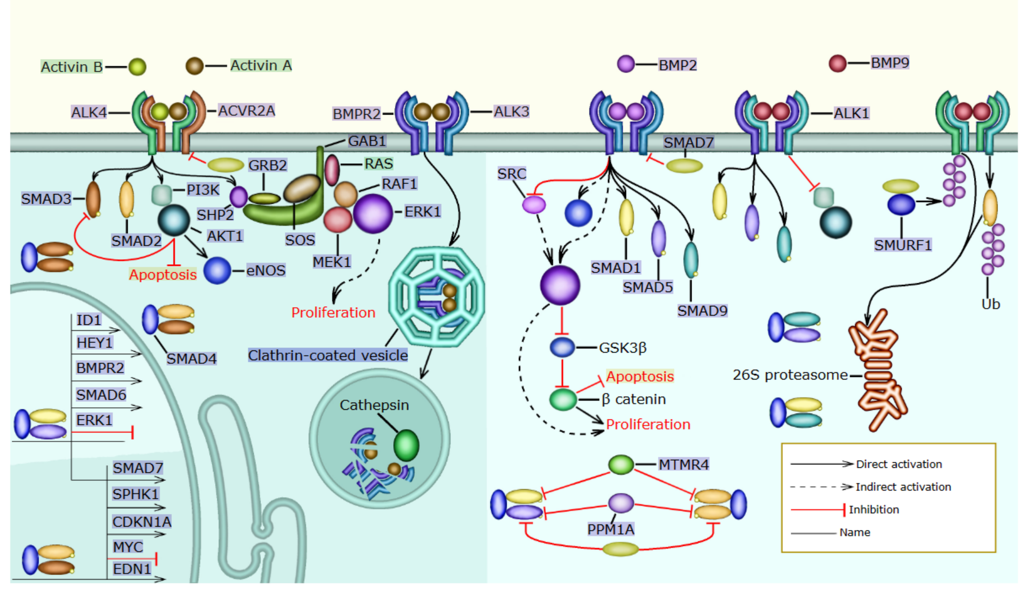

Yndestad, A. et al. Elevated levels of activin A in clinical and experimental pulmonary hypertension. J Appl Physiol 106, 1356–1364 (2009).

Ryanto, G. R. T. et al. An endothelial activin A-bone morphogenetic protein receptor type 2 link is overdriven in pulmonary hypertension. Nature Communications 2021 12:1 12, 1–14 (2021).

Humbert, M. et al. Sotatercept for the Treatment of Pulmonary Arterial Hypertension. New England Journal of Medicine 384, 1204–1215 (2021).

Created by Lifelike
lifelike.bio
